# Benefits and Risks of Melatonin in Hepatic and Pancreatic Disorders; A Review of Clinical Evidences

**DOI:** 10.22037/ijpr.2020.114477.14872

**Published:** 2021

**Authors:** Saeed Abdi, Mohammad Abbasinazari, Sara Ataei, Neda Khanzadeh-Moghaddam, Negin Keshvari

**Affiliations:** a *Gastroenterology and Liver Diseases Research Center, Research Institute for Gastroenterology and Liver Diseases, Shahid Beheshti University of Medical Sciences, Tehran, Iran. *; b *Department of Clinical Pharmacy, School of Pharmacy, Shahid Beheshti University of Medical Sciences, Tehran, Iran. *; c *Department of Clinical Pharmacy, School of Pharmacy, Hamadan University of Medical Sciences, Hamadan, Iran.*

**Keywords:** Melatonin, Liver, Pancreas, Review

## Abstract

Melatonin is the “clock factor” produced from the pineal gland dominating regular circadian rhythm in mammalians. It is an indoleamine with potent multifunctional pharmacological effects, both receptor dependent and non-receptor dependent effects, including antioxidant and anti-inflammatory activities. The aim of this review is to summarize clinical evidence related to melatonin’s effectiveness in the treatment of liver and pancreas diseases. Databases including PubMed, Scopus, and Cochran Library were searched up to November 2020.Finally, this review has summarized up-to-date clinical evidence to investigate the efficacy and safety of melatonin for the management of liver and pancreas diseases. Melatonin has been demonstrated to have beneficial effects on the management of Non-alcoholic fatty liver disease (NAFLD), sleep disturbance of cirrhotic patients, prevention of drug/poison induced liver toxicity,and prevention of post endoscopic retrograde cholangiopancreatography pancreatitis (PEP);more data is needed to recommend melatonin administration in the treatment of mentioned disorders.

## Introduction

Melatonin is the endogenous hormone responsible for different functions, including time regulation of circadian rhythms, stabilization of sleep/awake cycle, reduction of oxidative stress, increase in expression of antioxidant enzymes, impact on vascular constriction and dilation, modulation of immune function, and decrease of inflammation (1). Exogenous intake of oral melatonin can directly decrease oxidative stress by negating the hydroxyl radicals and plays an important role in inhibiting cell proliferation, inflammation, and apoptosis. Furthermore, melatonin can optimize the potential function of antioxidants enzymes such as glutathione peroxidase (GPx) and superoxide dismutase (SOD) indirectly (2). Former studies have suggested a positive impact of melatonin on improvement in hepatic and pancreatic disorders. However, there was no comprehensive review perusing the strength of the data that support this effect. On the other hand, there are some reports regarding the risks of melatonin in mentioned diseases. Therefore, the present review was conducted to assess the benefits and risks of melatonin in hepatic and pancreatic diseases.

## Experimental

PubMed, SCOPUS, and Cochrane library were used as the main electronic databases in this review to collect data, performing a systematic search of the articles published in the medical literature on the subject until November 2020. A commercially available software program (Endnote, Thomson Reuters, London, UK) was used for electronic title management. The combinations of keywords used for the electronic search were:

melatonin

melatonin and review

melatonin and liver

melatonin and “hepatic disease/disorders”

melatonin and pancreas

melatonin and “pancreatic disease/disorders”

melatonin and toxicity

In all, 12 eligible articles were identified, including nine original articles and three case reports. The obtained results were carefully evaluated by two specialists (a hepatologist and a clinical pharmacist), and the most important findings related to the use and effects of melatonin on hepatic and pancreatic diseases are summarized below.

## Results and Discussion

*1iver diseases*

Several cells have existed in the liver, including hepatocytes, cholangiocytes, Kup-ffer cells, hepatic stella cells, sinusoidal endothelial cells, and dendritic cells. However, factors causing damage to the liver tissue such as viruses, drugs, herbals, high-fat diet, and genetic mutations, and biliary tract diseases, ultimately progress into liver cirrhosis after activation of fibrogenesis. Current evidence shows that melatonin protects against liver injury by inhibiting oxidation, inflammation, hepatic stella cells proliferation, and hepatocyte apoptosis, thereby inhibiting the progression of liver cirrhosis (3). 

*Non-alcoholic fatty liver disease (NAFLD) and non-alcoholicsteato-hepatitis (NASH)*

Non-alcoholic fatty liver disease (NAFLD) is the most prevalent chronic liver disease, and non- alcoholic steato-hepatitis (NASH) is its advanced form. There are five eligible published articles regarding the effect of melatonin in NAFLD/NASH in the medical literature (4-8). In two trials effect of melatonin plus tryptophane versus placebo has been evaluated (4, 5); however, in three trials effect of melatonin versus placebo has been determined (6-8). The investigated studies were heterogeneous, although the heterogeneity sources were found by subgroup analysis. In addition, some confounding variables, such as weight, Body Mass Index (BMI), and gender were not controlled in the mentioned studies. The maximum and minimum duration of melatonin administration was four and 56 weeks, respectively. Based on this trials, melatonin in a dose of 6-18 mg/d can improve liver indices in NAFLD. Positive effects of melatonin were more in decrease level of Aspartate aminotransferase (AST), Alkaline phosphatase (ALP), and Gamma glutamyl transferase (GGT).However, none of the previous trials displayed any significant lowering effect of melatonin on level of Alanin aminotransferase (ALT).ALT level is often used to determine the hepatic inflammation and injury in NAFLD, but there are researches proved that ALT was normal in many NAFLD patients. In a meta-analysis, Ma et al.have demonstrated that ALT level is normal in 25% and 19% of NAFLD and NASH patients, respectively (10).They also have concluded that the value of ALT in the clinical diagnosis of NAFLD and NASH remains need to be further testified. The diagnosis of NAFLD requires the proof of an excessive accumulation of triglycerides in the hepatocytes by imaging or histology methods; simultaneously it is mandatory to exclude any significant alcohol consumption. Liver biopsy remains the gold standard for diagnosis and assessment of NAFLD. However many physicians prefer to use non-invasive methods (9). Hence, in most trials, such as mentioned, only biochemical parameters have been evaluated for diagnosis and follow up of the NAFLD/NASH patients (4-8). It seems that this is a limitation regarding trials investigating effect of an agent in management of NAFLD. Regarding safety of melatonin, no significant adverse effect was reported for melatonin or placebo in the trials (4-8).In the future doing more randomized clinical trials regarding the effect of melatonin in the treatment of NAFLD/NASH based on changes in histology of liver biopsy are recommended (instead of biochemical markers).

*Cirrhosis and related complications*

Disturbance in trace elements, vitamins, and endogenous transmitters has been reported frequently in cirrhotic patients (11). An imbalance in serotonin and melatonin homeostasis observed in patients with liver cirrhosis may be associated with advanced encephalopathy (12). Given that melatonin has antioxidative and anti‐inflammatory properties that effectively decrease liver injury, it is a potential agent with which to reverse liver cirrhosis in its early stage (3).

Given that alcohol‐induced liver cirrhosis is highly dependent on Reactive Oxygen System (ROS) related mechanism, melatonin has been shown an essential role in the inhibition of alcohol‐induced pathogenesis, including elevation of liver aminotransferases, triglyceride, and hepatic steatosis, by up‐regulating phosphorylation of AMP-activated protein kinaseand Superoxide Dismutase (SOD) in alcohol‐treated rats (13). Also, Current evidence suggests that melatonin may improve mesenchymal stem cells stemness or transplantation efficacy by enhancing antioxidant and anti‐inflammatory properties. It has been shown that combination of melatonin and mesenchymal stem cells suppressed liver fibrosis in mice and restored liver function significantly more than mesenchymal stem cells transplantation alone (14). 

There is not any human study regarding the effect of melatonin in cirrhosis development in the long term. At the moment, there is only one published trial regarding the melatonin effect in alleviation of sleep disorder in cirrhotic patients. Sleep disturbance are common among cirrhotic patients. Up to 40–50% of patients with cirrhosis complain from poor and unsatisfactory sleep (15). In a randomized, double-blind, placebo-controlled, cross-over clinical trial, cirrhotic patients (child A or B) with sleep disturbance, without hepatic encephalopathy, were randomized to melatonin (3 mg) or placebo for two weeks. Then patients have received a washout period of one week and crossed over to melatonin or placebo for a further two weeks. The Pittsburgh Sleep Quality Index (PSQI) and Epworth Sleepiness Scale (ESS) were used to measure sleep quality and daytime sleepiness, respectively. Patients who have received melatonin had a significantly lower PSQI and ESS compared to both pre-treatment (*P *< 0.001) and post placebo scores (*P* < 0.001). Side effects in two groups were similar (two each of abdominal pain, one each of headache, one each of dizziness). They have concluded that melatonin in a dose of 3 mg/d seems safe and effective for relief of sleep disturbance in the short term in cirrhotic patients (Child A or B) (16). However, the majority of researches regarding melatonin therapy are conducted in animals rather than human, leaving the therapeutic effects of melatonin on human cirrhosis currently undefined. In future doing more human trials regarding effect of melatonin in control of cirrhosis and or its complication is recommend.

*Drug/poison induced liver diseases*

Drug-induced liver injury (DILI) describes a spectrum of clinical manifestations and severity that ranges from a mild elevation of liver enzymes to acute liver failure and death. The most common offending agents are antimicrobials, herbal and dietary supplements (17). 

In a review article, Reiter *et al.* reported that melatonin reduces the toxicity and increases the efficacy of a large number of medications whose side effects are well documented. They have referred that the majority of these studies were done in animal models and a tiny number in the human (18). Only in one human study, protective effect against drug-induced hepatitis has been reported by melatonin in humans. The addition of melatonin (5 mg/d) with standard therapy in drug-induced hepatitis could result in 81.7% improvement versus 66.5% in standard therapy without using melatonin. Also, normalization of biochemical parameters was better in melatonin plus standard therapy versus standard therapy alone (19). Since there are limited human studies regarding the protective effect of melatonin against liver toxicity, the findings provide an additional benchmark for further studies involving more patients.

Although there is no published clinical report, several animal studies used melatonin as a protective agent against liver toxicity induced by mycotoxins, and promising results were reported. These properties can be related to the lipophilic nature of melatonin and its high tissue distribution. Direct antioxidant activity, anti-apoptotic activity via the reduction in caspase 3 and Heat Shock Protein 70 (HSP70) concentration, immunomodulatory activity via reduction of IL-1b, and finally down-regulation of cytochrome P450 enzyme in hepatocytes have been mentioned as mechanisms of melatonin against mycotoxin induced liver toxicity (20). Melatonin is a suitable candidate for future researches, including clinical trials against liver toxicity of mycotoxins.

*Autoimmune hepatitis (AIH)*

There are several published cases of the temporal relationship between melatonin or melatonin agonist use and the development or aggravation of autoimmune hepatitis (AIH). A case of AIH has been reported after beginning melatonin therapy to treat insomnia for the first time in 1997. Hepatic cell biopsy showed histologic features of AIH (21). Another case of AIH has been reported in a 50 years old man after starting ramelteon (melatonin agonist) who received relief from insomnia. Authors have concluded that the association between ramelteon use and the development of AIH suggests a role for ramelteon in the aetiology of his illness (22). A case of melatonin-induced biochemical flares in a patient with primary sclerosing cholangitis (PSC) with features of autoimmune hepatitis was reported in 2019. Following approximately seven months of successful management of PSC, the patient again started taking melatonin 5 mg nightly to improve sleep disorder. After about three weeks of starting melatonin, routine liver function tests revealed a marked elevation of serum alanine ALT and AST levels from values near normal to approximately ten and eight above upper limit standard, respectively. ALP and GGT levels also rose; however, they were significantly elevated before starting melatonin. The patient was strongly recommended to withdraw melatonin and avoid it in the future. The ALT, AST levels reduced significantly one month after melatonin withdrawal. Melatonin probably possesses important immunoenhancing activities, mostly underlined in immunosuppressed situations, and it may counteract the immune-depressive effect of glucocorticoids. Melatonin promotes a T helper1-mediated response (T helper-1 cells produce far more interferon γ (IFN-γ) and interleukin 2 (IL-2) than T helper 2 cells), thereby flare up inflammatory pathways (23). Although there is a limited case reports, it seems that melatonin and melatonin agonists must be prescribed with caution in patients with diagnosis of AIH. 

*Pancreas diseases*

Although melatonin has been recognized as the pineal hormone, several researches have shown that melatonin could synthesize indifferent tissues and that the gastrointestinal tract appears to be the main source of melatonin. Two main enzymes, involved in the control of melatonin production*, arylalkylamino-N-acetyl-serotonin-transferase* (AA-NAT), and *hydroxyindolo-O-methyl-transferase* (HIOMT) have been found in the gastrointestinal system. Also, recent studies have suggested that melatonin and its receptors are present in the pancreas (24).

*Acute pancreatitis*

The beneficial effect of melatonin on alleviation of acute pancreatitis is related to: 1) Increase in antioxidant properties of the pancreatic tissue, through direct scavenging of toxic radical oxygen (ROS) and nitrogen (RNS) species, 2) intensification of the activity of antioxidant enzymes, such as superoxide dismutase (SOD), catalase (CAT), or glutathione peroxidase (GPx), 3) the suppression of pro-inflammatory cytokine tumour necrosis α (TNFα) production, accompanied by stimulation of an anti-inflammatory IL-10, 4) increase in pancreatic blood flow and decrease in neutrophil infiltration, 5) decrease in apoptosis and necrosis in the inflamed pancreatic tissue, 6) elevation of chaperon protein (HSP60), and 7) promotion of regenerative process in the pancreas (24).

Chen *et al.* have mentioned that the low level of melatonin has been associated with an increased risk of acute pancreatitis (25). To our best knowledge, there is no published clinical trial regarding the effect of melatonin in the alleviation or prevention of acute pancreatitis. Several experimental researches have reported that the administration of melatonin to animal models prior to the induction of acute pancreatitis protected the pancreas from the development of acute inflammation and significantly diminished pancreatic tissue damage. Also the serum concentration of amylase and lipase (two important indicators of acute pancreatitis severity) and cytokine TNFα, were significantly decreased in the animals pre-treated with melatonin and subjected to acute pancreatitis. In contrast, the serum level of anti-inflammatory IL-10 was increased markedly in these animals (26). The findings provide an additional benchmark for further studies involving more patients. In future evaluation effect of melatonin in alleviation of acute pancreatitis as clinical trials recommend. 

*Post ERCP Pancreatitis (PEP)*

Endoscopic retrograde cholangio-pancreatography (ERCP) was developed as adiagnostic and therapeutic modality in pancreatobiliary diseases. Post Endoscopic retrograde cholangiopancreatography pa-ncreatitis (PEP) is the most prevalent complication of endoscopic retrograde. The guidelines of European Society of Gastro-intestinal Endoscopy recommend the routine administration of 100 mg of diclofenac or indomethacin immediately before or after ERCP to prevent PEP in all patients without contraindications to nonsteroidal anti-inflammatory drugs (NSAIDs) (27). Hernández-Velázquez *et al. *have evaluated the effects of peri-procedural administration of melatonin on the inflammatory response and lipid peroxidation associated with ERCP in a pilot study (n = 37). They have concluded that melatonin is safe and tolerable in patients undergoing ERCP, but it does not appear to affect inflammatory cytokine level or lipid peroxidation. Also the incidence of PEP was not reported in the mentioned trial (28). A recent study has been conducted to determine whether melatonin addition to indomethacin reduce the rate of PEP occurrence on 411 patients undergoing ERCP procedure. Participants were given oral melatonin (3 mg) plus indomethacin suppository (100 mg) or placebo plus indomethacin suppository (100 mg), one hour before doing the procedure. The results showed that the administration of melatonin plus indomethacin could decrease the PEP rate (9.3% versus 13.6% in Per-Protocol analysis) significantly (*P* = 0.034). Also, after 24 h, amylase and lipase levels were lower in the melatonin group than in the placebo group significantly (*P* = 0.041 and 0.032, respectively) (29).

**Table 1:** Summary of Clinical trials regarding effect of melatonin in liver/pancreas disorders

**Authors**	**Country**	**Year**	**Health condition**	**Number of patients**	**Type of the study**	**Blinding**	**Melatonin dose**	**Duration of follow up**	**Primary outcome**	**ref**
Celinski K *et al.*	Poland	2014	NAFLD^1^	56	RCT^2^	yes	10 mg/d	56 weeks	Melatonin is worth considering for the therapy of NAFLD , particularly in patients with impaired fat metabolism accompanied by hypertriglyceridemia and hyper-LDL cholesterolemia	4
Cichoz-Lach H *et al.*.	Poland	2010	NASH ^3^	30	RCT	yes	10 mg/d	4 weeks	Melatonin plus tryptophan have the significant impact on the reduction in plasma levels of pro-inflammatory cytokines and may be useful in the treatment of patients with NASH	5
Gonciarz M *et al.*	Poland	2012	NASH	42	RCT	yes	10 mg/d	24 weeks	Aspartate aminotransferase and gamma-glutamyl transpeptidase concentrations decrease significantly in Melatonin versus placebo group (*p* < 0.05, *p* < 0.05 respectively)	6
Pakravan H *et al.*	Iran	2017	NAFLD	97	RCT	yes	6 mg/d	6 weeks	In melatonin group, alanine aminotransferase after treatment were significantly decreased compare to baseline (*p* > 0.5) However aspartate aminotransferase did not change after treatment (*p* > 0.5)	7
Bahrami M *et al.*	Iran	2020	NAFLD	45	RCT	yes	18 mg/d	12 weeks	Melatonin had improvement effect on many factors related to NAFLD such as liver enzymes, hs-CRP, anthropometric measurements, blood pressure, leptin serum levels and the grade of fatty liver	8
De Silva AP *et al.*	Sri Lanka	2020	sleep disturbances in cirrhotic patients	71	RCT (crossover)	yes	3 mg/d	2 weeks, 1 week washout, 2 weeks	Melatonin is effective for relief of sleep disturbance in the short term in cirrhotic patients (Child A or B)	16
Propov SS *et al.*	Russia	2013	Patients under treatment of potentially hepatotoxic medications	54	Case-control	No	5 mg/d	4 weeks	Addition of melatonin with standard therapy in drug induced hepatitis could result to 81.7% improvement versus 66.5% in standard therapy without using melatonin	19
Hernández-Velázquez B *et al.*	Mexico	2016	Patients under ERCP^4^	37	RCT	yes	5 mg/d	1 week	Melatonin does not affect inflammatory cytokine level or lipid peroxidation after doing ERCP	28
Sadeghi A *et al.*	Iran	2019	Patients under ERCP	411	RCT	yes	3 mg/d	1 week	Administration of melatonin plus indomethacin could decrease the post ERCP pancreatitis rate significantly (9.3% *versus* 13.6%, *p* = 0.034)	29

NAFLD: Non Alcoholic Fatty Liver Disease; NASH: Non Alcoholic Steato-hepatitis; RCT: Randomized Clinical Trial; ERCP: Endoscopic Retrograde Cholangio-pancreatography.

## Conclusion

Table 1 represents the summary of nine eligible studies regarding the effect of melatonin in liver and pancreas disorders. This review found that there are few clinical trials regarding melatonin for treatment of liver and pancreas diseases so far. The available evidence is not sufficient to support the use of melatonin in clinical practice for this population. On the other hand, it seems that melatonin must be avoided in AIH until giving more information. Further research is still necessary to assess its effects (benefits and risks) for hepatic and pancreatic diseases.
